# *In Vivo* Monitoring of Multi-Unit Neural Activity in the Suprachiasmatic Nucleus Reveals Robust Circadian Rhythms in *Period1^−/−^* Mice

**DOI:** 10.1371/journal.pone.0064333

**Published:** 2013-05-22

**Authors:** Nana N. Takasu, Julie S. Pendergast, Cathya S. Olivas, Shin Yamazaki, Wataru Nakamura

**Affiliations:** 1 Laboratory of Oral Chronobiology, Graduate School of Dentistry, Osaka University, Suita, Osaka, Japan; 2 Department of Biological Sciences, Vanderbilt University, Nashville, Tennessee, United States of America; 3 Precursory Research for Embryonic Science and Technology, Japan Science and Technology Agency, Kawaguchi, Japan; Pennsylvania State University, United States of America

## Abstract

The master pacemaker in the suprachiasmatic nucleus (SCN) controls daily rhythms of behavior in mammals. C57BL/6J mice lacking *Period1* (*Per1^−/−^*) are an anomaly because their SCN molecular rhythm is weak or absent *in vitro* even though their locomotor activity rhythm is robust. To resolve the contradiction between the *in vitro* and *in vivo* circadian phenotypes of *Per1^−/−^* mice, we measured the multi-unit activity (MUA) rhythm of the SCN neuronal population in freely-behaving mice. We found that *in vivo Per1^−/−^* SCN have high-amplitude MUA rhythms, demonstrating that the ensemble of neurons is driving robust locomotor activity in *Per1^−/−^* mice. Since the *Per1^−/−^* SCN electrical activity rhythm is indistinguishable from wild-types, *in vivo* physiological factors or coupling of the SCN to a known or unidentified circadian clock(s) may compensate for weak endogenous molecular rhythms in *Per1^−/−^* SCN. Consistent with the behavioral light responsiveness of *Per1* ^−/−^ mice, *in vivo* MUA rhythms in *Per1* ^−/−^ SCN exhibited large phase shifts in response to light. Since the acute response of the MUA rhythm to light in *Per1^−/−^* SCN is equivalent to wild-types, an unknown mechanism mediates enhanced light responsiveness of *Per1^−/−^* mice. Thus, *Per1^−/−^* mice are a unique model for investigating the component(s) of the *in vivo* environment that confers robust rhythmicity to the SCN as well as a novel mechanism of enhanced light responsiveness.

## Introduction

The suprachiasmatic nucleus (SCN) is the master pacemaker in mammals that controls circadian rhythms in locomotor activity. Lesions of the SCN cause arrhythmicity of circadian rhythms [1]–[3] and transplants of fetal SCN tissue restore behavioral rhythms with the periodicity of the donor SCN [4]–[6]. A small number of rhythmic SCN cells is sufficient to drive behavioral rhythmicity, since the circadian locomotor activity rhythm was restored in hamsters by transplanting only a few SCN neurons [7]. *In vitro* monitoring of rhythms of neural activity and circadian gene expression has also demonstrated that the periods of rhythmic activity in SCN explants approximate the respective behavioral periodicities of wild-type and mutant rodents [8]–[13].

Together these studies have contributed to the conceptualization that SCN neurons, each having a cell-autonomous rhythm governed by molecular feedback loops, are coupled to each other resulting in an integrated period that drives the periodicity of the locomotor activity rhythm. Recent studies have identified exceptions to this tenet of mammalian circadian organization. For example, some mutant mice (Transgenic R6/2, *Fmr1^−/−^/Fxr2^−/−^*) have rhythmic SCN *in vitro*, but arrhythmic locomotor activity [14], [15]. In contrast, in C57BL/6J mice lacking the circadian gene, *Period1* (*Per1^−/−^* mice), circadian gene promoter activity (*Period1-luciferase*) and circadian gene fusion protein expression (PERIOD2::LUCIFERASE) in *Per1^−/−^* SCN explants are arrhythmic or have low-amplitude oscillations *in vitro*, while the wheel-running activity rhythm of *Per1^−/−^* mice is robust and indistinguishable from wild-types [16]. The *Per1-luciferase* rhythms in individual cells in *Per1^−/−^* SCN explants are severely compromised (most cells are arrhythmic), but a small subset of neurons retain rhythmicity. The phenotype of *Per1^−/−^* mice is perplexing because if the SCN molecular rhythm is necessary to drive rhythmic wheel-running activity, then how is it possible that *Per1^−/−^* mice have robust activity rhythms? One possibility is that those few neurons that retain molecular rhythms *in vivo* in the *Per1^−/−^* SCN are sufficient to drive behavior [7]. Consistent with this hypothesis, *Per1^−/−^* mice display large behavioral phase shifts in response to light pulses, suggesting that *Per1^−/−^* SCN have low-amplitude molecular rhythms [17]. Alternatively, *in vivo* physiological factors (which are not present in the *in vitro* culture) or coupling to a rhythmic oscillator may confer robust rhythmicity to the entire population of neurons in the *Per1^−/−^* SCN.

*In vivo* MUA (multi-unit activity) measures the ensemble electrical activity rhythm from a large population of SCN neurons and thus is ideal for investigating the *in vivo* phenotype of the *Per1^−/−^* SCN. In this study, we sought to resolve the contradiction between the *in vitro* and *in vivo* circadian phenotypes of *Per1^−/−^* mice by measuring MUA from the SCN of freely-behaving mice.

## Materials and Methods

### Ethics Statement

All experiments were conducted in accordance with animal protocols approved by the Animal Care and Use Committees at Osaka University (permission#AD-20-042-0) and Vanderbilt University (M/08/096). All surgery was performed under isoflurane anesthesia, and all efforts were made to minimize suffering.

### Animals

C57BL/6J (N10 to N11) heterozygous *mPer1^ldc+/−^* mice [16] were intercrossed to generate wild-type and *mPer1^ldc−/−^* mice that were genotyped as previously described [18]. For experiments measuring MUA, mice were bred and group-housed in the Osaka University Graduate School of Dentistry animal facility in a 12 h-light/12 h-dark cycle (12L∶12D) and provided food and water *ad libitum*. For experiments assessing the effects of ocular enucleation and activity feedback, mice were bred and group-housed in the Vanderbilt University animal facility in 12L∶12D and provided food and water *ad libitum*.

### Surgery and *in vivo* multi-unit neural activity recording

The experiments were performed according to the technique developed by Yamazaki et al. [19] for hamsters with minor modifications for mice [20], [21]. Bipolar electrodes were constructed from pairs of epoxy-coated stainless steel wires (bare diameter, 100 µm; tip distance, 75 µm; Unique Medical, Tokyo, Japan) and an uncoated platinum-iridium wire (diameter, 75 µm; A-M Systems, Sequim, WA, USA) was used as a signal ground in the cortex. Wires were connected to a 5-pin receptacle (1.25 mm pitch; Morex, Taipei Hsien, Taiwan) wrapped in insulated copper tape.

Male mice, 24–35 weeks of age and weighing 25–32 g, were anesthetized under isoflurane (1.5%; Abbott, Tokyo, Japan) and placed in a stereotaxic device (Narishige, Tokyo, Japan). The dorsal aspects of the parietal bones were exposed and cleaned. One hole was drilled around the bregma in the parietal bones. The electrodes were inserted into the brain, aimed at the SCN (0.4 mm posterior and 0.2 mm lateral to the bregma, 5.8 mm depth from the skull surface) and attached directly to the skull with orthodontic bond (3M Unitek, Monrovia, CA, USA).

After surgery mice were singly housed in an open-top cage (410 mm width×260 mm length×270 mm height) with a running wheel (170 mm diameter). The light intensity in the recording chamber was ∼100 lux at cage level (white LED, DL18E26D Denryo, Tokyo, Japan; mounted to the top of the light-tight box). The mice were allowed to recover from surgery for at least 1 week. Thereafter, the electrodes were connected to head stage buffer amplifiers (TL082; Texas Instruments, Dallas, TX, USA) located on the head of the mouse. Buffer amplifiers were connected to a slip ring (Biotex, Kyoto, Japan) that allowed free movement of the animal. Output signals were processed by a differential input integration amplifier (INA 101 AM; Burr-Brown, Tucson, AZ, USA; gain, ×10) and then fed into an AC amplifier (band-pass, 500 Hz to 5 kHZ; gain, ×10,000). Spikes were discriminated by amplitude and counted in 1-min bins using a computer-based window discrimination system (KPCI-1801HC; Keithley Instruments, Cleveland, OH, USA). The number of wheel-running revolutions was simultaneously recorded by the same computer-based system.

At the conclusion of the experiment, mice were anesthetized and positive current (1 µA; 60 s) was passed through the recording electrodes. Brains were removed and fixed in 4% paraformaldehyde in 0.1 M phosphate buffer containing 2% potassium ferrocyanide (Sigma-Aldrich, St. Louis, MO, USA). Serial coronal sections (25 µm thick) were stained with neutral-red and blue spots of deposited iron were identified as the recording site. Data were analyzed from mice that had both recording electrodes localized to one side of the SCN with one electrode in the ventral SCN and the other in the dorsal SCN, so that the rhythm was measured from the whole SCN. This electrode localization was confirmed in 4 wild-type mice (from a total of 8 mice) and 4 *Per1^−/−^* mice (from a total of 24 mice).

### Light responsiveness experiments

The onset of activity [circadian time (CT) 12] was determined for 3 d in constant darkness (DD) and linear regression (ClockLab, Actimetrics, Wilmette, IL, USA) was used to predict the onset of activity (CT12) on the day of the light pulse. At CT15, the mouse received a 15-min light pulse (intensity 100 lux, white LED as described above for recording chamber). To determine the phase shift in locomotor activity, one regression line was fit to the onset of activity for 3 d before the light pulse and the second line was fit to the onset of activity for 4 d following the light pulse (excluding the first cycle immediately after the pulse) and the phase shift was calculated that took into account the change in period that occurred with the light pulse (ClockLab). The phase shifts in the MUA rhythms were analyzed similarly except that acrophase was used as the phase marker for the MUA rhythm (ClockLab). Mean (±SD) MUA responses of the SCN to light pulses were plotted relative to baseline, which was determined from the mean counts 15 min before the light pulse and set to 100% in wild-type and *Per1* ^−/−^ mice. The number of units recorded varies from mouse to mouse so the absolute baseline is different for each animal. Thus, the relative MUA response must be determined for each animal. The relative MUA response during the light pulse was determined for each mouse by dividing the average relative MUA response (%) during the 15 min light pulse by the average MUA (%) during the 15 min before the light pulse. Then the relative MUA responses during the light pulse were averaged for each genotype.

### Ocular enucleation experiments

Male *Per1^−/−^* mice (n = 2; 12 weeks of age) were singly housed in cages (140 mm width×330 mm length×170 mm height) with unlimited access to a running wheel (110 mm diameter), food, and water. The cages were placed in light-tight, ventilated boxes in l2L∶12D (light intensity: 200–300 lux). Cages were changed every 3 weeks. Wheel-running activity (recorded every minute by computer) was monitored using ClockLab. Mice were anesthetized under isoflurane anesthesia and Buprenex (1 mg/kg) was administered subcutaneously immediately before bilateral ocular enucleation. After surgery, mice were returned to their cages and wheel-running was recorded in 12L∶12D for 2 days and then in DD. Wheel-running activity data were double-plotted in actograms in 5-minute bins using ClockLab.

### Activity feedback experiments

Male *Per1^−/−^* mice (n = 5; 8–22 weeks of age) were singly housed in cages (140 mm width×330 mm length×170 mm height) with locked wheels (wheels were present in the cages but they could not rotate), food, and water. The cages were placed in light-tight, ventilated boxes in l2L∶12D (light intensity: 200–300 lux) for 7 days and then the mice were released into DD. Cages were changed every 3 weeks. General activity was monitored every minute with passive infrared sensors [22] and data were double-plotted in actograms in 5-min bins using ClockLab. χ^2^ periodogram analysis (p<0.001) were performed on days 1–14 in DD to determine if significant rhythmicity was present.

### Data analysis

Actograms of wheel-running and multi-unit neural activity were generated using ClockLab analysis software. Cosinor analyses of MUA rhythms were performed using Circadian Physiology software (v2.3; http://www.circadian.org/softwar.html) [23]. Cosine curves were fit to the data (3 cycle in LD, 3 cycles in DD) in 6-min bins (all data fit with p<0.000001) using periods ranging from 22 to 26-h by 0.1-h steps. For the best period, the mesor and amplitude were determined. To determine the phase [in Zeitgeber time (ZT), where ZT0 is lights on and ZT12 is lights off) of the MUA rhythm in LD, data were fit with a cosine curve with a fixed 24-h period. The period of the wheel-running rhythm was determined by linear regression (ClockLab).

Means were statistically compared (SPSS Statistics software, IBM, Armonk, NY, USA) with independent samples *t* tests (two-tailed), with the following exceptions. If the variances were not homogeneous, then a Welch's t-test was used. If the data were not normally distributed (determined by the Sapiro-Wilk test), then the non-parametric Mann-Whitney U test was used. The detection of statistically different differences is limited by n = 4/group. Significance was ascribed at *p*<0.05. Results are expressed as the mean ± SD.

## Results

### *In vivo* MUA measures the rhythm of the SCN neuronal population

**Figure 1:**
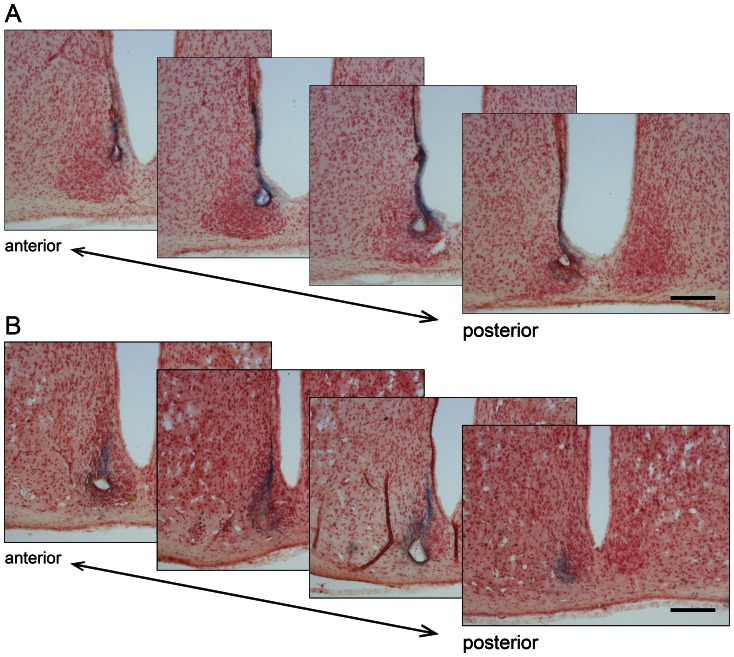
Histological examination of recording sites in the ventral and dorsal SCN. Representative serial coronal sections (25 µm, every other section shown) of a wild-type (A) and *Per1^−/−^* (B) SCN following MUA recording. Sections were stained with neutral-red and blue spots of deposited iron are the recording sites. Recording sites were present in the ventral and dorsal SCN. The anterior to posterior orientation of the sections is indicated. Scale bar represents 200 µm. The data in A and B correspond to the data shown in Fig. 2A and B, respectively.

We first measured the rhythm from a large, diverse population of SCN neurons by differential recording from two bipolar electrodes, one electrode implanted in the ventral (core) region and the other electrode in the dorsal (shell) region of wild-type and *Per1^−/−^* SCN. Histological examination of each SCN after recording showed that one electrode was placed in the ventral (core) region and the other in the dorsal (shell) region of the SCN in wild-type (Fig. 1A) and *Per1^−/−^* mice (Fig. 1B). These data demonstrate that MUA rhythms were measured from the whole SCN in all animals examined.

### *In vivo* MUA rhythms in *Per1^−/−^* SCN are robust

**Figure 2:**
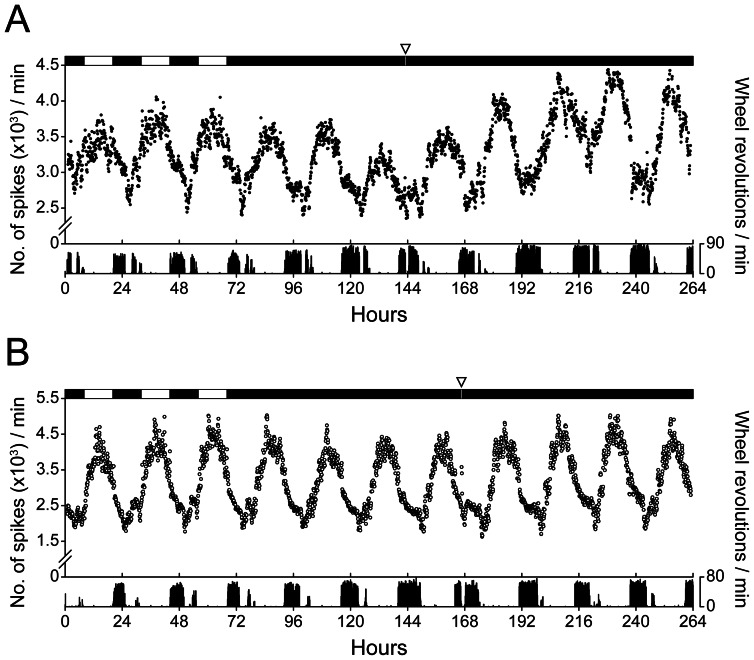
The *in vivo* circadian rhythm in the multi-unit neural activity of *Per1* ^−/−^ SCN is similar to wild-type SCN. Representative serial-plotted actograms (6-min bins) of multi-unit neural activity (top trace; y-axis: spikes/min) recorded from wild-type (A; black circles) and *Per1^−/−^* (B; open circles) SCN in freely-behaving mice. The bottom trace is simultaneously-recorded wheel-running activity (y-axis: wheel revolutions/min). Open bars are light and closed bars are dark (top of figure). A 15-min light pulse (indicated by open inverted triangle; 100 lux) was administered during the early subjective night (CT15). The number of spikes (for MUA) or wheel revolutions were counted every minute and integrated in 6-min bins.

To assess the *in vivo* phenotype of the *Per1^−/−^* SCN, we measured MUA from freely moving wild-type (Fig. 2A) and *Per1^−/−^* (Fig. 2B) mice in the light-dark cycle (LD) and in constant darkness (DD). *In vivo*, the amplitude of neural activity in *Per1^−/−^* SCN was indistinguishable from wild-types in LD and DD (Table 1), indicating that the many neurons in the *Per1^−/−^* SCN are rhythmic. The phases of the MUA rhythms in LD did not differ between wild-type (ZT: 6.25±0.31) and *Per1^−/−^* (ZT: 6.49±0.89) mice (*t*-test, *p* = 0.628). The period of the MUA rhythm in DD was slightly, but statistically significantly, longer in *Per1^−/−^* SCN compared to wild-type SCN (Table 1).

**Table 1:** Comparison of circadian rhythm parameters in wild-type and *Per1^−/^* ^−^ mice.

Parameter	wild-type	*Per1^−/−^*	*p*-value
MUA in LD			
Mesor (spikes/min)	4339±2351	6781±6466	0.773###
Amplitude (spikes/min)	1415±1119	1258±717	0.820#
Period (h)	23.93±0.39	24.18±0.56	0.491#
MUA in DD			
Mesor (spikes/min)	4171±1968	6615±6475	0.686#
Amplitude (spikes/min)	1150±825	1085±509	0.898#
Period (h)	23.60±0.14	23.85±0.13	0.040#
Wheel-running period (h)	23.80±0.23	23.87±0.12	0.885###
Light pulse at CT15			
Relative MUA during light pulse (%)	124±18	117±19	0.579#
Phase shift in wheel-running activity	−1.50±0.84	−3.10±0.21	0.028##
Phase shift in MUA	−1.59±0.30	−3.00±0.42	0.002#

Cosine curves were fit to the multi-unit (MUA) neural activity data recorded from wild-type or *Per1^−/−^* SCN in the light-dark cycle (LD; 3 d) or in constant darkness (DD; 3 d) using periods ranging from 22 to 26-h by 0.1-h steps. For the best period, the mesor and amplitude were determined. Fifteen-min light pulses (100 lux) were administered at CT15 (3 h after activity onset). The relative MUA response during the light pulse was determined for each mouse by dividing the average relative MUA response (%) during the 15 min light pulse by the average MUA (%) during the 15 min before the light pulse. Then the mean relative MUA responses during the light pulse were averaged for each genotype. To determine the magnitudes of the phase shifts, one regression line was fit to the onset of activity or acrophase of the MUA rhythm for 3 d before the light pulse and the second line was fit to the 4 d following the light pulse (excluding the first cycle immediately after the pulse) and the phase shift was calculated that took into account the change in period that occurred with the light pulse. Data are the mean ± SD; *n* = 4 wild-type and n = 4 *Per1* ^−/−^ mice.

# Student's t-test,

## Welch's t-test,

### Mann-Whitney U test.

### Robust circadian locomotor activity rhythms persist in *Per1^−/−^* mice after ocular enucleation and removal of activity feedback

**Figure 3:**
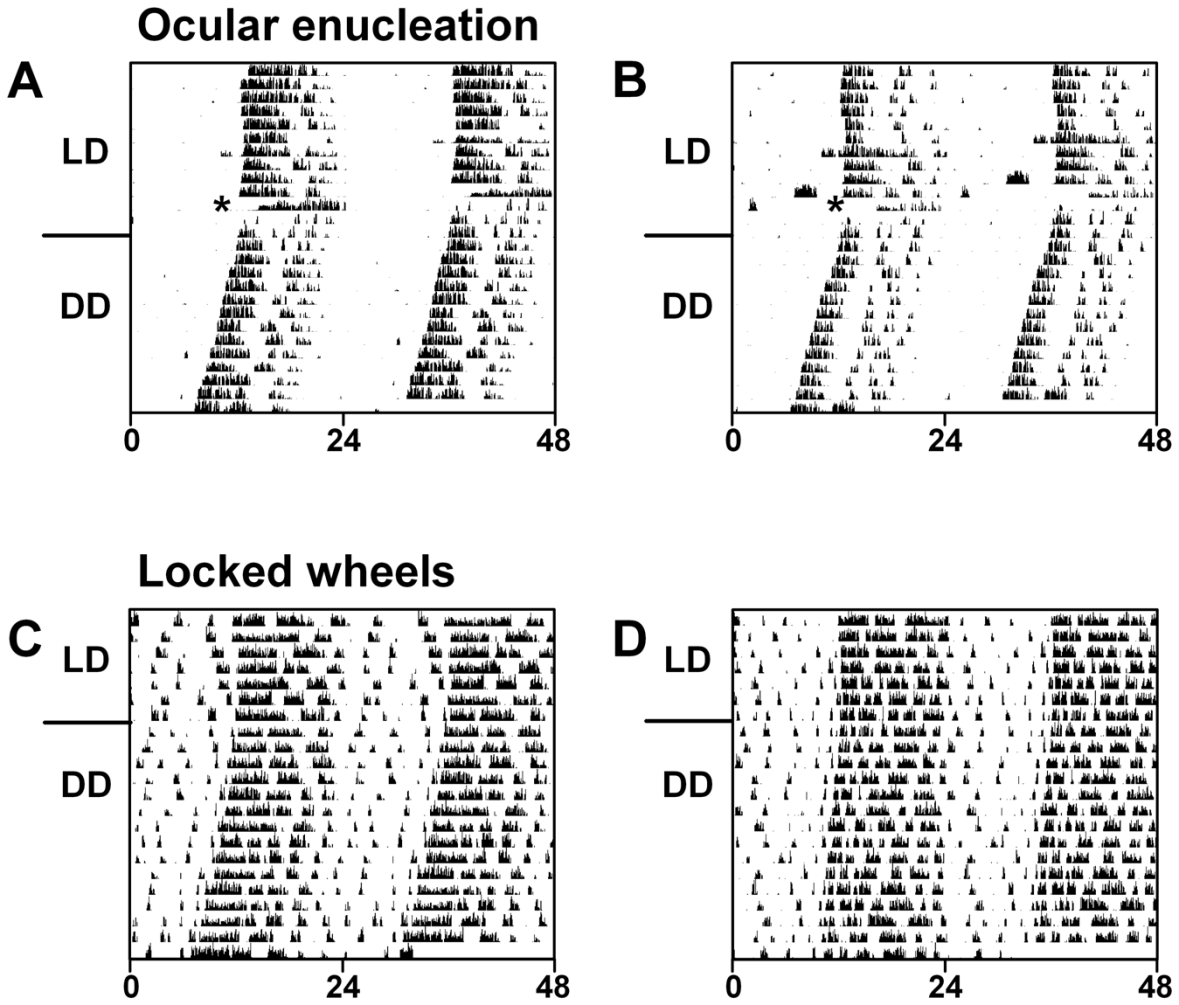
Robust locomotor behavior rhythms in *Per1^−/−^* mice persist after ocular enucleation and in the absence of wheel-running activity feedback. Double-plotted actograms (5-min bins) of locomotor activity of male *Per1^−/−^* mice (x-axis: time in hours; y-axis: days). **A, B.** Wheel-running activity was recorded from *Per1^−/−^* mice (n = 2) maintained in 12L∶12D (LD) and ocular nucleation was performed (indicated by asterisks). Two days after surgery, mice were released into constant darkness (DD). **C, D.** *Per1^−/−^* mice (n = 5) were housed in cages with locked running wheels (wheels were present but could not rotate), maintained in 12L∶12D and then released into DD. General activity was continuously monitored with passive infrared sensors.

To determine if robust oscillations were conferred to the *Per1^−/−^* SCN by coupling of the SCN with the circadian clock in the retina, we recorded wheel-running activity after ocular enucleation of *Per1^−/−^* mice (Fig. 3A, B). We found that robust circadian rhythms of wheel-running persisted after ocular enucleation, suggesting that *Per1^−/−^* SCN rhythmicity is not conferred by coupling to the retina clock.

It has previously shown that SCN MUA is altered by running wheel activity [19], [21], [24]. To determine if activity feedback to the SCN contributed to robust SCN rhythms in *Per1^−/−^* mice, we analyzed general activity of *Per1^−/−^* mice housed with locked running wheels (wheels were present in the cage but could not rotate; Fig. 3C, D). We found that circadian locomotor activity rhythms of *Per1^−/−^* mice were robust in LD and DD in the absence of activity feedback (χ^2^ periodogram detected significant rhythmicity in all *Per1^−/−^* mice after ocular enucleation).

### Light responsiveness of the SCN in *Per1^−/−^* mice

**Figure 4:**
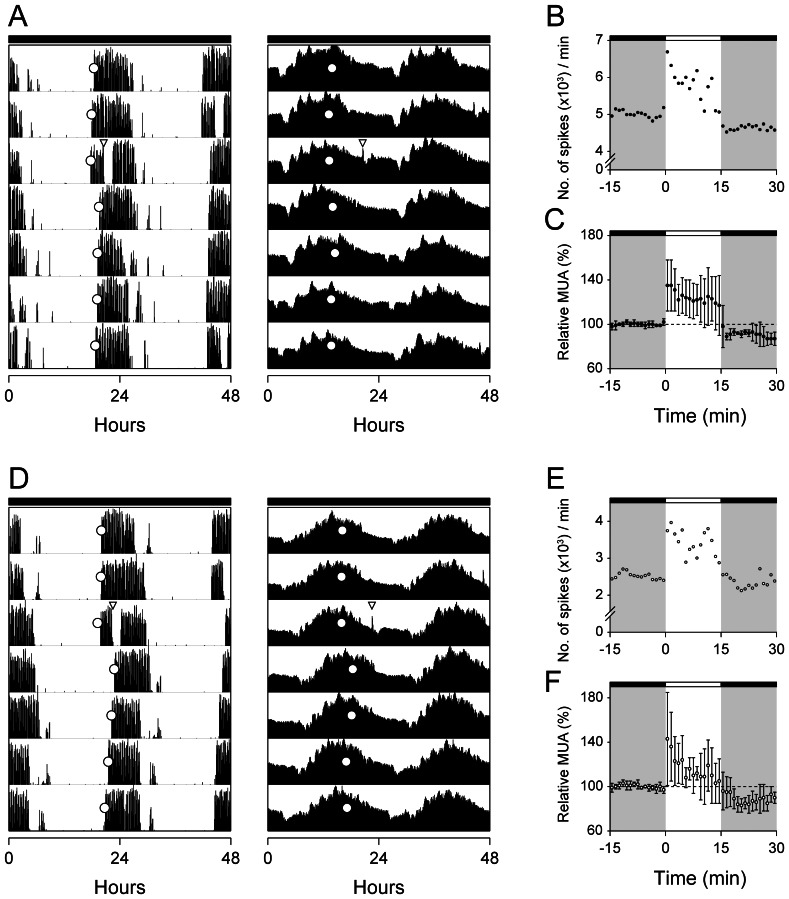
Behavioral and SCN responses to light. Representative double-plotted actograms of wheel-running activity (left panels) and multi-unit neural activity in the SCN (right panels) of wild-type (A) and *Per1* ^−/−^ (D) mice. Each line represents two consecutive days (48 hr), with time in 6-min bins plotted left to right. Consecutive days are aligned vertically. The mice were maintained in constant darkness (indicated by the black bars above actograms) and a 15-min light pulse (100 lux) was administered 3 hr after activity onset (CT15; open inverted triangle). Open circles indicate the respective phase markers: activity onset for wheel-running activity and acrophase for MUA. The magnitudes of phase shifts were determined by measuring the phase differences between least-squares fitted regression lines through the respective phase markers before and after the light pulse [locomotor: −2.20 h, MUA: −1.94 h in (A); locomotor: −3.24 h, MUA:−2.60 h in (D)]. To show the acute response of the SCN to light, MUA data (plotted in 1-min bins) surrounding the light pulse in (A) and (D) are magnified in (B) and (E), respectively. The time of the light pulse is indicated by the open bars at the tops of the figures and dark is indicated by gray shading. Mean (±SD) MUA responses in wild-type (C; n = 4) and *Per1^−/−^* (F; n = 4) SCN are shown relative to baseline (baseline determined from the mean counts 15 min before the light pulse and set to 100% in wild-type and *Per1* ^−/−^ mice).

*Per1^−/−^* mice exhibit large phase shifts (∼4 h) in behavior in response to light pulses administered during the early subjective night [17]. To assess the response of the *Per1^−/−^* SCN to light, we simultaneously measured MUA in the SCN and wheel-running activity in wild-type (Fig. 4A) and *Per1^−/−^* (Fig. 4D) mice administered 15-min light pulses at CT15. We found that the acute increase in MUA and the duration of the acute increase in the SCN in response to the light pulse did not differ between wild-type (Fig. 4B, C) and *Per1^−/−^* (Fig. 4E, F) mice (Table 1: Mean relative MUA during light pulse). The magnitudes of the phase shifts in the SCN and wheel-running activity rhythms were greater in *Per1^−/−^* mice compared to wild-types (Table 1). In both genotypes, the magnitude of the phase shift in the MUA rhythm matched that of the phase shift in behavior.

## Discussion

C57BL/6J *Per1^−/−^* mice are unique because their circadian behavioral phenotype, which is indistinguishable from wild-types, is not predicted from their arrhythmic (or weakly rhythmic) *in vitro* SCN phenotype. This discrepancy between the *in vivo* behavioral and *in vitro* SCN phenotypes raises intriguing questions regarding the mechanisms whereby the SCN controls circadian behavior in these mice. Is the ensemble of neurons in *Per1^−/−^* SCN rhythmic *in vivo*? Does a physiological factor(s) present in the *in vivo* environment confer high-amplitude oscillations to the *Per1^−/−^* SCN? Is a robust rhythm imparted to the *Per1^−/−^* through coupling with an extra-SCN oscillator? To address these questions, we examined the *in vivo* phenotype of the *Per1^−/−^* SCN by simultaneously monitoring MUA in the SCN and wheel-running activity in freely-behaving *Per1^−/−^* mice. We found that *in vivo Per1^−/−^* SCN had robust, high-amplitude MUA rhythms in the light-dark cycle and in constant darkness that were similar to wild-type mice. The MUA rhythm we measured reflects the neural activity of a large population of SCN neurons because we obtained differential recordings from electrodes implanted in the ventral and dorsal SCN. Thus, the high-amplitude MUA rhythm in the *Per1^−/−^* SCN demonstrates that the ensemble of neurons, rather than a few rhythmic neurons, is driving robust locomotor activity in *Per1^−/−^* mice. Similar to a previous study demonstrating that the age-related decline in the amplitude of the locomotor activity rhythm was reflected in the amplitude of the *in vivo* MUA SCN rhythm (but not the rhythm of PER2::LUC expression in *ex vivo* SCN), this study demonstrates that *in vivo* MUA is a reliable predictor of circadian behavior [20].

These data suggest that some component of the *in vivo* environment confers robust rhythmicity to the *Per1^−/−^* SCN. Our finding that the *in vivo* environment may be pro-rhythmic is reminiscent of previous studies that demonstrated differences between the *in vitro* and *in vivo* effects of tetrodotoxin (TTX) on the SCN rhythm-inhibition of action potentials with TTX had marked effects on the maintenance of the SCN oscillation *in vitro* [25] but seemingly not *in vivo* [26].

Physiological factors, such as temperature and hormones, are candidates for modulating *in vivo* rhythmicity. Alternatively, a rhythmic circadian clock may exist in *Per1^−/−^* mice that couples to the SCN, resulting in high-amplitude SCN MUA rhythms. The retina is one such candidate oscillator, as our previous study suggested that coupling of the retina to the SCN stabilizes the free-running period of locomotor activity in hamsters [27]. However, we found that robust circadian activity rhythms persisted in *Per1^−/−^* mice after ocular enucleation, suggesting that coupling to the retina clock does not confer robust rhythmicity to the *Per1^−/−^* SCN. Consistent with our result, it was recently demonstrated that *Per1^−/−^* retinas are arrhythmic *in vitro* [28], thus the retina is not a likely candidate as a rhythmic circadian clock in *Per1^−/−^* mice.

Since all peripheral tissues in *Per1^−/−^* mice that have been examined are arrhythmic *in vitro* [9], [16], perhaps an unidentified circadian clock, whose rhythm persists without *Per1*, couples to the SCN [29]. It is also possible that the *Per1^−/−^* SCN may be coupled to a rhythmic oscillator that is closely apposed to the SCN. Coronal 300 µm *Per1^−/−^* SCN explants are arrhythmic *in vitro*, but we cannot rule out the possibility that a nucleus just rostral or caudal to the SCN confers robust rhythmicity to the SCN *in vivo* (a thicker coronal section of the SCN cannot be examined because it will not survive in culture).

Physical activity induced by running wheel availability, forced treadmill running, or drug administration shifts the phase of rodent circadian rhythms and free-running periods of circadian rhythms are shortened in the presence of a running wheel [30]–[34]. We have previously demonstrated that wheel-running activity feeds back to the SCN and decreases its neural activity in hamsters and mice [19], [24] thus it is possible that wheel-running activity drives robust MUA rhythms in the *Per1^−/−^* SCN. However, we found that robust locomotor activity rhythms persist in *Per1^−/−^* mice housed with locked running wheels, suggesting that wheel-running activity feedback is not responsible for robust SCN rhythms in *Per1^−/−^* mice.

*Per1^−/−^* mice exhibit large behavioral phase shifts in response to light pulses administered during the subjective night [17]. A previous study demonstrated that the augmented light responsiveness of *Clock* mutant (Δ19) heterozygous mice is attributed to their low-amplitude SCN rhythms [35]. In contrast to *Clock* mutant mice, we found that *Per1^−/−^* SCN have high-amplitude MUA rhythms, suggesting that the enhanced light responsiveness of *Per1^−/−^* mice cannot be attributed to the amplitude of the rhythm in the SCN. However, a recent study demonstrated that coupled limit cycle oscillators may behave differently from single oscillators such that the inverse relationship between MUA amplitude and phase-shifting capacity may not hold true for neuronal networks [36]. It is also possible that the *in vivo* MUA rhythm does not reflect the amplitude of the molecular timekeeping rhythm, such that the molecular rhythm could be low-amplitude while the MUA rhythm is robust. The relationship between the amplitude of the molecular rhythm and the amplitude of the electrical activity rhythm in the SCN should be further examined.

Large behavioral phase shifts may also occur if the *Per1^−/−^* SCN has enhanced sensitivity to light. If so, then the acute MUA response to light pulses would be greater in *Per1^−/−^* SCN compared to wild-types. However, we found that the magnitude and duration of the acute MUA responses of the wild-type and *Per1^−/−^* SCN to light were identical to each other, suggesting that they have equivalent sensitivities to light. Alternatively, it is possible that the sensitivity of the molecular timekeeping rhythm is altered in *Per1^−/−^* SCN, which could then account for enhanced light responsiveness of *Per1^−/−^* mice.

Together these data suggest that the high-amplitude rhythm of the *Per1^−/−^* SCN is conferred by the *in vivo* environment, resulting in robust wheel-running activity of *Per1^−/−^* mice. Whether a physiological factor or coupling to another oscillator (or both) confers rhythmicity to the *Per1^−/−^* SCN is unknown. Furthermore, the enhanced light responsiveness of *Per1^−/−^* mice cannot be attributed to either a low-amplitude MUA rhythm in the SCN or to enhanced responsiveness of the MUA rhythm in the SCN to light. Thus, the mechanism of enhanced light responsive in *Per1^−/−^* mice is unknown, but does not appear to operate at the level of the MUA rhythm. Future studies of C57BL/6J *Per1^−/−^* mice are necessary to elucidate the *in vivo* physiological factor or unidentified circadian clock(s) that compensates for weak endogenous rhythms in *Per1^−/−^* SCN and to identify the mechanism of enhanced responses to light.
